# The role of *ESR1* in regulating lactation traits in buffalo mammary epithelial cells

**DOI:** 10.5194/aab-69-347-2026

**Published:** 2026-06-25

**Authors:** Zhixiang Wang, Xiaoyun Chen, Chaobin Qin, Jiaqi Jiang, Ling Li, Qingyou Liu, Cuijin Teng, Jinping Xiong, Leyi Wang, Ahmed Amin, Zhipeng Li

**Affiliations:** 1 Guangxi Key Laboratory of Animal Reproduction, Breeding and Disease Control, College of Animal Science and Technology, Guangxi University, Nanning, 53004, China; 2 Guangxi Zhuang Autonomous Region Buffalo Milk Quality and Safety Control Technology Engineering Research Center, Guangxi Buffalo Research Institute, Chinese Academy of Agricultural Sciences, Nanning, 530001, China; 3 Guangdong Provincial Key Laboratory of Animal Molecular Design and Precise Breeding, School of Life Science and Engineering, Foshan University, Foshan, 528225, China; 4 Royal Cell Biological Technology (GUANGXI) Co. Ltd., Nanning, 530007, China; 5 Animal Production Department, Faculty of Agriculture, Cairo University, Cairo, 1263, Egypt

## Abstract

Buffalo milk is favoured by consumers due to its higher nutritional components, as well as its rich and mellow flavour. However, research on the molecular mechanisms underlying the formation of lactation traits in buffalo remains limited. In this study, we systematically investigated the regulatory effects of estrogen receptor alpha (*ESR1*) on lactation-related functions in buffalo mammary epithelial cells (BMECs) using overexpression and small interfering RNA (siRNA)-mediated silencing strategies. Our results demonstrated that *ESR1* knockdown via siRNA significantly suppressed BMEC viability and proliferation and downregulated the expression of genes associated with lipid transport and secretion (*PPARG, LPL, PLIN2, VLDLR, ADFP, CD36*), milk protein synthesis (*CSN1S1, CSN1S2, RPS6, RHEB, EIF4E, AKT1*), and cell proliferation (*STAT5A, MTOR, ELF5, CCND1, EGF, IGF1*). Concurrently, silencing *ESR1* markedly upregulated pro-apoptotic genes (*BAX*, *CASP9*) and downregulated the anti-apoptotic gene *BCL2*. Conversely, *ESR1* overexpression exerted opposite effects on BMECs, further validating the critical role of *ESR1* in modulating BMEC proliferation and milk component synthesis. In conclusion, *ESR1* expression enhances buffalo lactation traits by promoting milk fat and milk protein synthesis, stimulating BMEC proliferation, and inhibiting cellular apoptosis. These findings provide novel insights into the molecular mechanisms governing buffalo lactation and offer valuable references for the development and utilization of lactation-related genetic resources in buffalo breeding.

## 1 Introduction

Milk has long been recognized as a primary source of essential human nutrition, supplying critical macronutrients and micronutrients – including fats, proteins, vitamins, and minerals – that support fundamental biological processes. Beyond its nutritional value, milk and dairy products are pivotal in global food security and agricultural economic sustainability. Among dairy livestock, the water buffalo (*Bubalus bubalis*) ranks as the second most important milk-producing species worldwide, surpassed only by cattle (Khetra et al., 2022). Notably, buffalo milk is distinguished by its high concentrations of fat, protein, and bioactive compounds, making it exceptionally valuable for manufacturing high-quality dairy products such as cheese and butter (Nie et al., 2022). Despite its economic and nutritional importance, buffalo milk production faces persistent challenges. Buffalo breeds across Asia and Africa – regions housing the majority of the global buffalo population – exhibit lower milk yield, shorter lactation duration, longer calving intervals, and slower genetic improvement relative to dairy cattle (Mohammad et al., 2018). A major limiting factor is the insufficient understanding of the genetic basis and molecular regulatory networks governing buffalo lactation, largely due to the scarcity of targeted mechanistic studies.

Lactation is a highly coordinated, complex biological process governed by intricate molecular networks. It encompasses mammary gland development; mammary epithelial cell metabolism, proliferation and differentiation; and milk component synthesis and secretion, all under precise regulation by numerous coding and non-coding genetic elements (Hannan et al., 2023; Lu et al., 2021). Among several factors affecting mammary gland development, estrogen receptor alpha (ESR1) has been considered to be a key regulator of epithelial cell proliferation, differentiation, and their functions. By binding to estrogens such as estradiol, *ESR1* directly influences the lactation process (Rusidzé et al., 2021). Thus, *ESR1* expression and activity have been linked to the initiation, maintenance, and cessation of lactation (Arendt and Kuperwasser, 2015; Schams et al., 2003). Moreover, previous studies have revealed that *ESR1* contributes to mammary duct growth, morphogenesis, and milk yield in cattle. For instance, a genome-wide association study conducted in Vrindavan crossbred cattle provides a significant association between the *ESR1* gene and milk production and lactation traits such as total milk yield, lactation length, and peak yield (Gangwar et al., 2025). Lactation is a highly coordinated, complex biological process governed by intricate molecular networks. It encompasses mammary gland development, mammary epithelial cell metabolism, proliferation and differentiation, and milk component synthesis and secretion, all under precise regulation by numerous coding and non-coding genetic elements (Fan et al., 2021). In mouse models, *ESR1* deletion causes severe defects in mammary duct morphogenesis and terminal lobule development (Bocchinfuso et al., 2000). Investigations conducted in Hu sheep have revealed that *ESR1* expression reaches its peak during puberty compared to lower levels in adulthood, indicating its crucial involvement in the mammary gland development of this species (Zhang et al., 2019). *ESR1* is a crucial transcriptional regulator controlling several signalling pathways and genetic networks linked to mammary epithelial cell biology. Specifically, estrogen-mediated *ESR1* signalling has been demonstrated to govern cellular proliferation, differentiation, and metabolic functions not only through classical genomic actions but also via the activation of rapid signalling cascades, such as the PI3K/AKT-mTOR pathway (Levin, 2009; Marino et al., 2006). Similarly, treatment with the anti-estrogen tamoxifen in heifers also resulted in a significant reduction in *ESR1* expression and hindered mammary parenchymal development, demonstrating that *ESR1* is a crucial receptor mediating estrogen-promoted mammary duct elongation and branching (Han et al., 2026; Tucker et al., 2016).

While the role of *ESR1* in orchestrating the regulation of genes critical for controlling mammary epithelial cell growth, proliferation, and apoptosis has been extensively investigated in several animals such as cattle, sheep, and mice (Connor et al., 2005; Mallepell et al., 2006; Zhang et al., 2019), its specific functions in buffalo lactation remain largely uncharacterized. Accordingly, this study aimed to elucidate how *ESR1* regulates genes governing BMEC proliferation, apoptosis, and the synthesis of major milk components (milk fat and protein) using siRNA silencing and lentiviral overexpression systems. Our findings are expected to deepen the understanding of the molecular regulation of BMEC function during lactation and support the development of genetic and management strategies to improve buffalo milk production.

## 2 Materials and methods

### 2.1 Experimental design

In order to explore the functional role of *ESR1* in mammary epithelial cell proliferation and its regulatory impact on milk synthesis and lactation in buffalo, mammary epithelial cells were obtained, isolated, cultured, and characterized to verify their epithelial origin and viability. The siRNA molecule was then specifically created to target *ESR1* mRNA in the epithelial cells. At the same time, a lentiviral *ESR1* overexpression-specific vector was constructed. Either siRNA or the lentiviral vector was then introduced into the cultured cells to attain either* ESR1* gene silencing or overexpression. Following that, the epithelial cells' transfection efficiency was evaluated to confirm the successful delivery of the constructed molecules. To explore the transcriptional regulatory role of *ESR1* in milk synthesis and lactation traits, the expression activity of key genes associated with milk fat transport and secretion was evaluated using qRT-PCR. Furthermore, cell proliferation was assessed using CCK-8 and 5-Ethynyl-2^′^-deoxyuridine (EdU) assays to validate the functional effects of *ESR1* gene expression modulation on the tested cells.

### 2.2 Laboratory animals and sampling

The buffalo mammary gland tissues utilized in this research were obtained from the Buffalo Breeding Farm of the Buffalo Research Institute, Chinese Academy of Agricultural Sciences (Nanning, Guangxi, China). All animals were managed under identical feeding and housing conditions. Murrah buffaloes aged 5–6 years with second or third parity were enrolled in this study. All experimental animals were in the mid-lactation stage ( 100±20 d in milk) and the diestrous stage during sample collection and experimentation. To collect mammary gland tissue samples, the buffaloes were transported to a local slaughterhouse and electrocuted using high-pressure electricity. The experimental protocol received approval from the Ethics Committee for Animal Experiments at Guangxi University (approval no. GXU-2024-094).

### 2.3 Buffalo mammary epithelial cell isolation, culture, and characterization

BMECs were isolated, referring to a previous study (Jiang et al., 2025). Briefly, fresh mammary gland tissues were immediately harvested from the buffaloes at a local slaughterhouse. Visible extraparenchymal adipose and connective tissues were carefully dissected away to isolate the central secretory parenchyma of the mammary gland. The obtained parenchymal tissues were rinsed three times with phosphate-buffered saline (PBS) containing 400 $\mathrm{IU}\;{\mathrm{mL}}^{-1}$ penicillin–streptomycin and then were transported to the laboratory within 2 h in sterile 0.9 % normal saline maintained at 38 °C . Upon laboratory arrival, the tissue samples were subjected to sequential rinsing with PBS containing penicillin–streptomycin at a descending concentration gradient (2000, 1200, and 400 $\mathrm{IU}\;{\mathrm{mL}}^{-1}$ ) to guarantee sterility prior to epithelial cell isolation. Thereafter, the tissues were finely cut into approximately 1 ${\mathrm{mm}}^{3}$ explants using sterile surgical scissors. These tissue explants were seeded into culture flasks and incubated at 38.5 °C in Dulbecco's Modified Eagle Medium/Nutrient Mixture F-12 (DMEM/F12) supplemented with 16 % fetal bovine serum (FBS) and 1 % penicillin–streptomycin (Gibco, Thermo Fisher Scientific, CA, USA) for 7 d. Fibroblast contamination was removed using the differential adhesion method. Mixed primary cells were digested with 0.25 % trypsin-EDTA (Gibco) for 3 min; the supernatant containing detached fibroblasts was discarded. The remaining adherent BMECs were digested for an additional 5 min, harvested, and centrifuged at 250 g for 3 min. After three rounds of differential purification, highly pure BMECs were obtained for identification and functional assays. Cell morphology and growth were monitored routinely. To confirm epithelial identity, total RNA was extracted from BMECs and buffalo fibroblasts (BFFs, negative control) using TRIzol reagent and then reverse-transcribed to cDNA. The relative expression of BMEC marker genes (*LALBA*, *CSN2*, *CSN3*) was quantified by qRT-PCR using gene-specific primers (Table 1).

**Table 1:** Primers used in this study for qRT-PCR.

Genes	Primer sequences( $5^{\prime }-3^{\prime }$ )	Access no.	Tm (°C)
*ESR1-Exon5*	F:TTCTTCTCCTCAAGCAGGTGG	XM_025294352.2	59.65
	R:GGCTTGGATCTGGTGTAGCA		59.75
*PPARG-Exon6*	F:CTCCACAGTTCAGCCCACAT	XM_044933439.2	59.96
	R:ACGACGACGGAGTAACTTGC		60.39
*LPL-Exon5-6*	F:CCGCAGACAGGATTACAGGAGGA	NM_001290913.1	63.64
	R:CAGTTAGCCACAGATTCGGTCACTC		63.34
*VLDLR-Exon14-15*	F:GTGGCGGAATTGGCAACATAAGAA	XM_025281566.3	62.4
	R:ACAGGTCCTCTTCAGTGGTCTTCAA		63.4
*PLIN2-Exon3*	F:GGTCTGAAGTAGGCAGGCAG	XM_044939804.1	60.11
	R:ATGTGCGAGTTCTGGACAGG		60.04
*CD36-Exon4-5*	F:GGTGATGAGAAGGCGGAAATGTTCA	NM_001290838.1	63.55
	R:CACACCAACACTGAGCAAGACGAT		63.57
*ACACA-Exon 38*	F:TAGATAGCCACGCAGCCACACT	XM_025281124.3	63.76
	R:GGTCCATCACCACAGCCTTCAT		63.3
*ADFP-Exon4*	F:TCGCAATGCTGCCTCCTTTTA	XM_044939806.1	60.61
	R:CTGAAATCAAGTGCCAGCCAG		59.8
*RPS6-Exon5*	F:ACTATACCAGGCGTCTGCAC	XM_006070892.3	59.54
	R:CGGGAAAGAGATGTTCAGCTTC		59.32
*CSN1S1-Exon9*	F:TGTTTGGAAAGGATATTGGGAGTG	XM_006071126.3	58.98
	R:GTAACGCTCAGAGGGCACAT		60.11
*CSN1S2-Exon7*	F:TGGCCATTCATCCCAGCAAG	XM_044945499.1	60.69
	R:GAGCTGTTCTCTGTTCAGTGC		59.2
*RHEB-Exon2*	F:TGGTGGGGAAAGTACAAATACC	XM_025291862.3	57.96
	R:TCTCTTCTACGCCTGGACTT		57.78
*EIF4E-Exon2-3*	F:CAGGAGGTTGCTAACCCAGA	NM_001319800.1	59.02
	R:GGATATGGTTGTACAGAGCCCA		59.56
*AKT1-Exon3*	F:CACCTGACCAAGACGACAGC	XM_025270896.3	60.95
	R:TCCCAGTCCTCGGAAGCAT		60.31
*PDK1-Exon6*	F:AGTTATTAAAGCAAAATCACCAGGA	XM_044933762.2	57.22
	R:TGCTCTCATTGCATTCTTGAAAA		57.54
*STAT5A-Exon16*	F:TGTACCCGCAGAACCCTGAC	XM_006055752.4	61.82
	R:GAGAGGGGTCCAGACTGTCC		60.97
*MTOR-Exon41*	F:TGGGGACTGCTTTGAGGTTG	XM_025285881.2	60.18
	R:GTGTGGCACGTGATCCTGTA		60.04
*ELF5-Exon4*	F:AAACCTCCTCTTTGGACCCAGC	XM_006062381.4	62.48
	R:CCAGTAGGAGTCGCAAGCTGTT		62.45
*CCND1-Exon2*	F:ATCAGATGTGACCCGGACTG	XM_006072357.4	59.17
	R:TCAGATGTTCACGTCACGCA		59.97
*EGF-Exon5*	F:TTGGTGCGATACCAAGCAGT	XM_045166310.1	59.96
	R:CACGGCAAATGAGTGACCTAC		59.27
*IGF1-Exon3*	F:TGCTTTTGTGATTTCTTGAAGGTGA	XM_006058813.4	59.87
	R:AGAGCATCCACCAACTCAGC		60.04
*BAX-Exon3*	F:GCTCTGAGCAGATCATGAAGACAG	XM_006050927.4	61.27
	R:GCTCAGCTTCTTGGTGGATGC		61.88
*BCL2-Exon3*	F:GAGCAGCTCTAACTGGAGAGT	XM_025273635.3	58.9
	R:ACCTCCTCCGTGATGTTGTA		58.07
*CASP9-Exon4*	F:CTCCTCCTCAGGGTCGCTAA	XM_006072791.4	60.4
	R:GCAGGAAGGTTTCAGGGTGA		59.89
*CASP3-Exon5*	F:TTTCATTATTCAGGCCTGCCG	XM_025280224.2	58.98
	R:GCGTTTCGCCAGGAAAAGTA		59.13
*LALBA-Exon3*	F:TCAGTTTGCCTGAATGGGTCT	NM_001290936.1	59.57
	R:TGCTTGAGTGAGGGTTCTGG		59.6
*CSN2-Exon4*	F:AAGCCTTTCAAGCAGTGAGGA	NM_001290879.1	59.86
	R:ATCCTGGAGTTCATCCTCTGTT		58.54
*CSN3-Exon4*	F:GCAAGAGCTGACGGTCACAA	XM_006071122.3	60.88
	R:GCTCCTGGGCACCCAAAAAA		61.41
*GAPDH-Exon 7*	F:TGGGTGTGAACCACGAGAAG	XM_006065800.4	59.89
	R:CGTGGACGGTGGTCATAAGT		59.75

### 2.4 siRNA synthesis and transfection

Two *ESR1*-targeting siRNA sequences and a negative control (siRNA-NC) were designed and synthesized by Sangon Biotech (Shanghai, China) based on buffalo *ESR1* mRNA (XM_025294352.2) (Table 2). BMECs were seeded in six-well plates and cultured to 70 %–80 % confluence prior to transfection. For each well, 100 pM siRNA and 5 µL transfection reagent (Sangon Biotech) were diluted separately in 100 µL Opti-MEM, mixed gently to form complexes, incubated at room temperature for 20 min, and then added dropwise to cells. The transfection efficiency was evaluated via a two-step complementary evaluation strategy. First, successful cellular delivery of the recombinant vectors was qualitatively verified by detecting green fluorescence signals under an inverted fluorescence microscope. Second, and more critically, the actual interference or overexpression efficiency was quantitatively determined by quantifying the mRNA expression level of the target ESR1 gene using qRT-PCR. All transfections were performed in triplicate, with non-targeting siRNA-NC as the control.

**Table 2:** The sequences of siRNA fragments targeting *ESR1* used in this study.

siRNA	Sense ( $5^{\prime }-3^{\prime }$ )	Antisense ( $5^{\prime }-3^{\prime }$ )
siRNA1	GGAGAAUGUUGAAGCACAA	UUGUGCUUCAACAUUCUCC
siRNA2	GCUACUGUGCAGUGUGCAA	UUGCACACUGCACAGUAGC
siRNA-NC	UUCUCCGAACGUGUCACGUTT	ACGUGACACGUUCGGAGAATT

### 2.5 Construction of lentiviral overexpression vector and infection

The* ESR1* gene was cloned from buffalo mammary gland tissues and inserted into the phBLV-CMV-MCS-3Flag-EF1-ZsGreen-T2A-Puro vector. Positive clones were sent to Sangon Biotech (Shanghai, China) for sequencing, and plasmids with correct sequences were extracted for further use. Subsequently, 293T cells were seeded into cell culture dishes at a density of approximately $1\times 10^{7}$ cells per dish. When the cell confluency reached approximately 70 %–80 %, 10 µg of the constructed ESR1-hBLV-CMV-MCS-3Flag-EF1-ZsGreen-T2A-Puro plasmid, 10 µg of the viral packaging auxiliary plasmid pSAX2, and 5 µg of the viral packaging auxiliary plasmid pMD2G were mixed according to the manufacturer's instructions. After incubation for 15 min, the mixture was slowly added to the 293T cells, which were then cultured in the incubator (37 °C , 5 % ${\mathrm{CO}}_{2}$ ). A total of 16 h after the transfection, the medium was replaced with fresh complete medium containing 10 % FBS (Gibco, Thermo Fisher Scientific, CA, USA). Viral supernatants were collected twice at 48 and 72 h post-transfection, respectively. Viral supernatants in the 50 mL centrifuge tubes were centrifuged at 4 °C , 2000 g for 10 min to remove cell debris. The supernatant of the viral stock was then collected and transferred to an ultracentrifuge tube, followed by ultracentrifugation at 4 °C , 82 700 g for 120 min. The viral pellet was completely resuspended in medium, and the ultracentrifuged suspension was aliquoted into sterilized viral storage tubes and stored at -80°C until further use. The MOI (multiplicity of infection) of the lentivirus was tested, and the MOI=100 showed an applicable concentration (Fig. S1 in the Supplement). After a 24 h infection period, the virus-containing medium was replaced with fresh complete culture medium. The transfection efficiency was also assessed via a two-step complementary evaluation strategy as described above. The successful cellular delivery was verified by detecting green fluorescence signals 48 h after infection under an inverted fluorescence microscope. Total RNA was extracted from the cells 72 h after infection, and the overexpression efficiency was determined by qRT-PCR. Each infection was performed in triplicate, and BMECs infected with a non-targeting (empty) lentiviral vector served as negative controls (LVG-NC).

### 2.6 Total RNA extraction and single-stranded cDNA synthesis

In this study, total RNA was extracted using the Trizol method. Briefly, BMECs or tissues were collected and supplemented with 1 mL of Trizol reagent (Abclonal, Shanghai, China). The cells were then transferred to 1.5 mL Eppendorf tubes, vigorously shaken, and lysed on ice for 5 min. Subsequently, 200 µL of chloroform was added, followed by vigorous shaking for 30 s and incubation on ice for 5 min. The lysate was then centrifuged at 12 000 g for 10 min at 4 °C . The supernatant was carefully transferred to a new RNase-free tube. After that, an equal volume of pre-cooled isopropanol was added, gently mixed, and incubated for 2 min at 4 °C . After centrifugation at 12 000 g for 5 min, the supernatant was discarded, and the RNA pellet was washed with 1 mL of pre-cooled 70 % ethanol twice. The supernatant was removed, and the tube was then transferred to a sterile container and incubated for 3–5 min to allow for ethanol evaporation. The RNA precipitate was subsequently dissolved using 20 µL DEPC-treated water. RNA quality was assessed using a NanoDrop ND-3000 spectrophotometer (Thermo Fisher Scientific, Waltham, MA, USA) and 1.5 % agarose gel electrophoresis. Only RNA samples exhibiting an A260 / 280 ratio between 1.8 and 2.0 and distinct ribosomal bands with no detectable degradation on the gel were selected for further study. For cDNA synthesis, 1 µg total RNA from each sample was used. Genomic DNA was eliminated using DNase I, and the first-strand cDNA was synthesized using the RevertAid First Strand cDNA Synthesis Kit (K1622; Thermo Fisher Scientific). Synthesized cDNA was stored at -80°C until further use.

### 2.7 Gene expression analysis using quantitative real-time PCR

Specific primers of target genes (Table 1) used in this study were designed using Oligo 7 software (Zhu et al., 2023) and synthesized by GenSys Biotech (Nanning, China). The qRT-PCR reactions were set up in 20 µL , containing 10 µL of 2× SYBR qPCR Premix (Abclonal, Shanghai, China), 2 µL of single-stranded cDNA template, 0.6 µL of a forward and reverse primer mixture (0.3 µM), and 7.4 µL of deionized water. qRT-PCR was performed using a Roche fluorescence PCR instrument (Shanghai, China). Relative expressions of each mRNA were analysed using the $2^{-\Delta \Delta \text{Ct}}$ method, and *GAPDH* was used as the endogenous control gene.

### 2.8 Cell proliferation rate assessment by using the CCK-8 method

BMECs were seeded in 96-well plates for 24 h before experimentation, with a precise cell count of approximately 20 000 cells per well to guarantee consistency. Each experimental group consisted of three replicates. Upon reaching a cell proliferation density of 60 %–70 % following overnight incubation, BMECs were transfected with siRNA or LVG vector, and cell proliferation was assessed at 24, 48, and 72 h post-transfection, respectively. Subsequently, following the manufacturer's guidelines, culture medium (Gibco, Thermo Fisher Scientific, CA, USA) was replaced with a fresh batch, supplemented with 10 µL of CCK-8 reagent (Abclonal, Shanghai, China). After incubation at 37 °C for 2 h, the absorbance (OD) at a 450 nm wavelength of each well was measured by a versatile microplate reader (Infinite M200PRO TECAN, Tecan, Switzerland).

### 2.9 Cell proliferation rate assessment by using the 5-Ethynyl-20-Deoxyuridine (EdU) method

BMECs were plated in triplicate in 48-well plates at a density of $2\times 10^{4}$ cells per well and incubated overnight at 37 °C with 5 % ${\mathrm{CO}}_{2}$ . Upon reaching a proliferation density of 60%∼70% , cells were transfected with the siRNA or lentiviral overexpression vector. Subsequently, the cells were exposed to 10 µm EdU (Beyotime, Shanghai, China) for 4 h in the dark. Following this, the cells were fixed in 4 % paraformaldehyde in PBS (Beyotime, Shanghai, China) for 20 min at room temperature and subjected to three 5 min washes with 1 mL of immunostaining blocking solution (Beyotime, Shanghai, China). Each well was then treated with 1 mL of immunostaining permeant (Beyotime, Shanghai, China) at room temperature for 15 min. The click reaction solution was prepared as per the provided instructions, with 0.5 mL applied to each well. After a 30 min incubation at room temperature in the dark, cells were washed twice with PBS (containing 400 $\mathrm{IU}\;{\mathrm{mL}}^{-1}$ penicillin and streptomycin). Subsequently, cells were incubated with Hoechst33342 (Beyotime, Shanghai, China) for 10 min at room temperature in the dark, followed by washing twice with PBS . Images were captured using an EVOS microscope (Thermo Scientific, CA, USA). The proliferation rate was calculated as the percentage of EdU-positive cells relative to total Hoechst-positive cells using ImageJ software.

### 2.10 Statistical analysis

Gene expression levels were quantitatively analysed using the $2^{-\Delta \Delta \text{Ct}}$ method, with significant differences between groups assessed via Student's t -test. Epithelial quantification in mammary tissue was conducted utilizing ImageJ software and GraphPad Prism version 8.1 (GraphPad Software, La Jolla, CA, USA) for comprehensive analysis and visualization. Statistical significance was denoted as follows: * for p<0.05 , ** for p<0.01 , and *** for p<0.001 .

## 3 Results

### 3.1 Isolation, culture, and identification of BMECs

**Figure 1:**
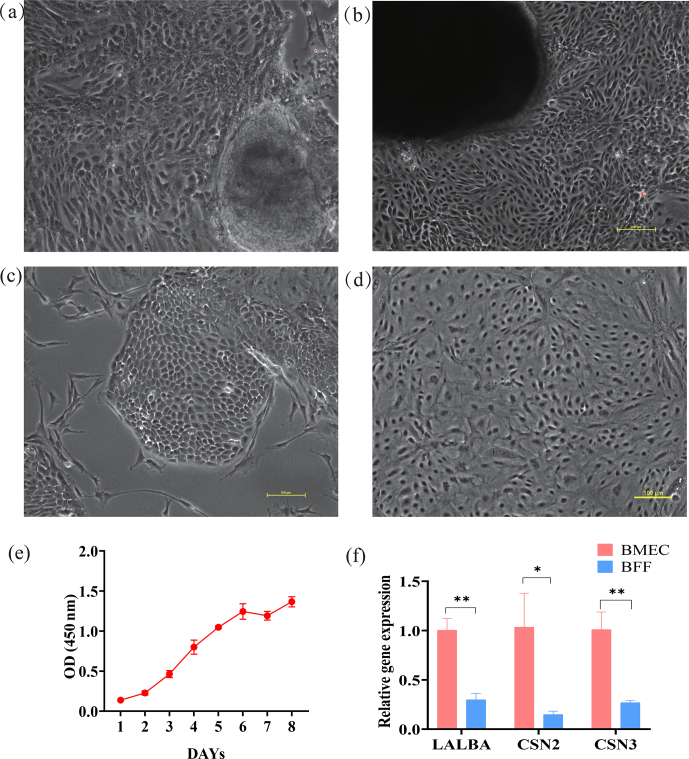
Isolation, culture, and identification of buffalo mammary epithelial cells (BMECs). **(a, b)** The BMECs on day 1 and day 14; Scale=100µm . **(c)** Unpurified BMECs; Scale=100µm . **(d)** Purified BMECs; Scale=100µm . **(e)** Growth curve of BMECs. **(f)** qRT-PCR analysis of the expression levels of BMECs' specific marker genes. Note: *, **, *** represent p<0.05 , p<0.01 , p<0.001 , respectively.

After 2 weeks of isolation and culture of mammary tissues, BMECs were observed migrating out from the tissues (Fig. 1a and b). Among the isolated cells, fibroblasts exhibited a spindle-shaped or irregular morphology, while mammary epithelial cells showed an oval-like shape (Fig. 1c). The purified mammary epithelial cells showed good condition (Fig. 1d). In addition, the proliferation rate of mammary epithelial cells was relatively slow for the first 2 d (lag phase), entered the rapid growth phase (logarithmic phase) on days 3–5, and reached an 80 % confluent state (plateau phase) after day 6, with the growth pattern conforming to the growth curve (Fig. 1e). qRT-PCR analysis revealed that the expression levels of BMECs' specific marker genes, including *CSN2*, *CSN3*, and *LALBA* in the isolated BMECs are significantly higher than those in BFFs. Together with the morphological characteristics, it can be confirmed that the isolated cells are BMECs.

### 3.2 siRNA transfection downregulates the expression levels of *ESR1* and inhibits the BMECs' proliferation

**Figure 2:**
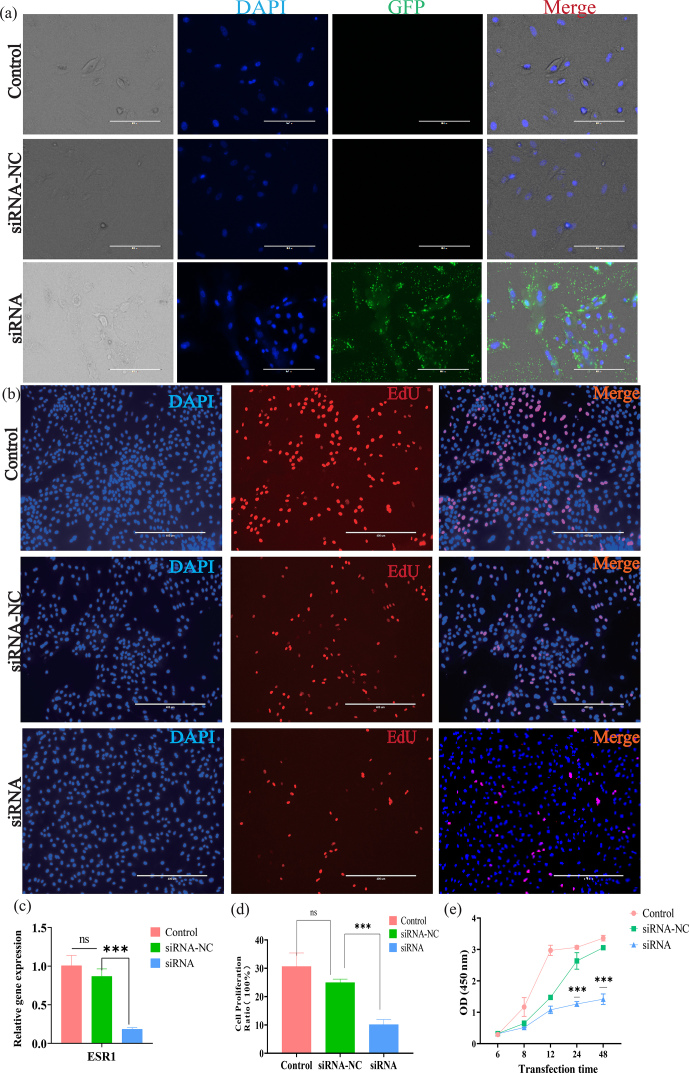
The effect of *ESR1* interference on the proliferation of buffalo mammary epithelial cells (BMECs). **(a)** Fluorescence detection of BMECs after *ESR1* siRNA interference; Scale=200µm . **(b)** EdU detection of BMECs proliferation after *ESR1* siRNA interference; Scale=400µm . **(c)** Expression of *ESR1* after RNA interference. **(d)** Statistical data of EdU detection. **(e)** CCK-8 detection of the proliferation of BMECs after *ESR1* RNA interference. Note: *, **, and *** represent p<0.05 , p<0.01 , and p<0.001 , respectively.

Following siRNA transfection targeting *ESR1* into BMECs, green fluorescence was observed within the cells (Fig. 2a), indicating successful siRNA delivery into BMECs. To confirm the siRNA interference effects on the *ESR1* mRNA abundance, qRT-PCR analysis was performed in the transfected and control groups of cells. Results showed that the siRNA interference significantly downregulated the expression levels of *ESR1* (Fig. 2c), verifying the silencing efficacy of the siRNA. Moreover, the EdU assay was used to evaluate the viability of BMECs, and our results revealed a significant decrease in proliferative activity in BMECs following the siRNA interference transfection (Fig. 2b and d). Consistently, the CCK-8 assay showed that, compared to the control group, the OD values in the *ESR1* interference group were significantly decreased at 24 and 48 h, indicating that suppression of *ESR1* expression markedly inhibits the proliferation of BMECs.

### 3.3 *ESR1* suppression via siRNA interference modulates the expression levels of milk-synthesis- and secretion-related genes in BMECs

**Figure 3:**
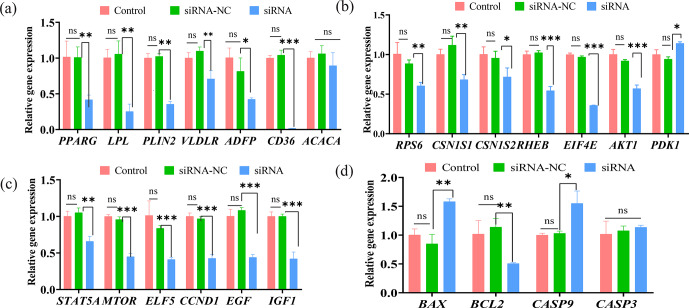
The effect of *ESR1* interference on gene expression in buffalo mammary epithelial cells (BMECs). **(a)** The effect of *ESR1* siRNA interference on the expression of genes related to milk fat synthesis. **(b)** The effect of *ESR1* siRNA interference on the expression of genes related to milk protein synthesis. **(c)** The effect of *ESR1* siRNA interference on the expression of genes related to cell proliferation. **(d)** The effect of *ESR1* siRNA interference on the expression of genes related to cell apoptosis. Note: *, **, and *** represent p<0.05 , p<0.01 , and p<0.001 , respectively.

qRT-PCR was performed to investigate the effect of *ESR1*-specific siRNA interference in BMECs on the transcription activities of milk synthesis and secretion traits. Results showed that the expression levels of *PPARG* (peroxisome proliferator-activated receptor gamma), *LPL* (lipoprotein lipase), *PLIN2* (perilipin 2), *VLDLR* (very low-density lipoprotein receptor), *ADFP* (adipose differentiation-related protein), and *CD36* (cluster of differentiation 36) were significantly downregulated after siRNA transfection into BMECs compared to the control group. However, the expression levels of *ACACA* (acetyl-CoA carboxylase alpha) did not exhibit a significant change (Fig. 3a). Moreover, the expression levels of milk protein synthesis-related genes, including *CSN1S1* (casein alpha-S1 chain) and *CSN1S2* (casein alpha-S2 chain), as well as key genes of the *MTOR* signalling pathway, including *RPS6* (ribosomal protein S6), *RHEB* (Ras homolog enriched in brain), *EIF4E* (eukaryotic initiation factor 4E), and *AKT1* (protein kinase B alpha, PKB α ), were also significantly reduced; however, the expression level of *PDK1* (3-phosphoinositide-dependent protein kinase 1) was increased (Fig. 3b). In addition, following *ESR1* siRNA interference, the expression levels of *STAT5A* (signal transducer and activator of transcription 5A), *MTOR* (mammalian target of rapamycin), *ELF5* (E74-like factor 5), *CCND1* (cyclin D1), *EGF* (epidermal growth factor), and *IGF1* (insulin-like growth factor 1) were significantly downregulated compared to the control group (Fig. 3c). *BAX* (BCL-2-associated X protein) and *CASP9* (Caspase-9) apoptotic genes exhibited elevated expression levels compared to the non-treated control group, while the expression level of *BCL2* (B-cell lymphoma 2 protein) was significantly decreased (Fig. 3d). These results suggested that *ESR1* siRNA interference can inhibit lipid transport and secretion, suppress protein synthesis in BMECs, and inhibit the proliferation and promote the apoptosis of BMECs.

### 3.4 *ESR1* overexpression upregulates the expression levels of *ESR1* and improves the BMECs' proliferation

**Figure 4:**
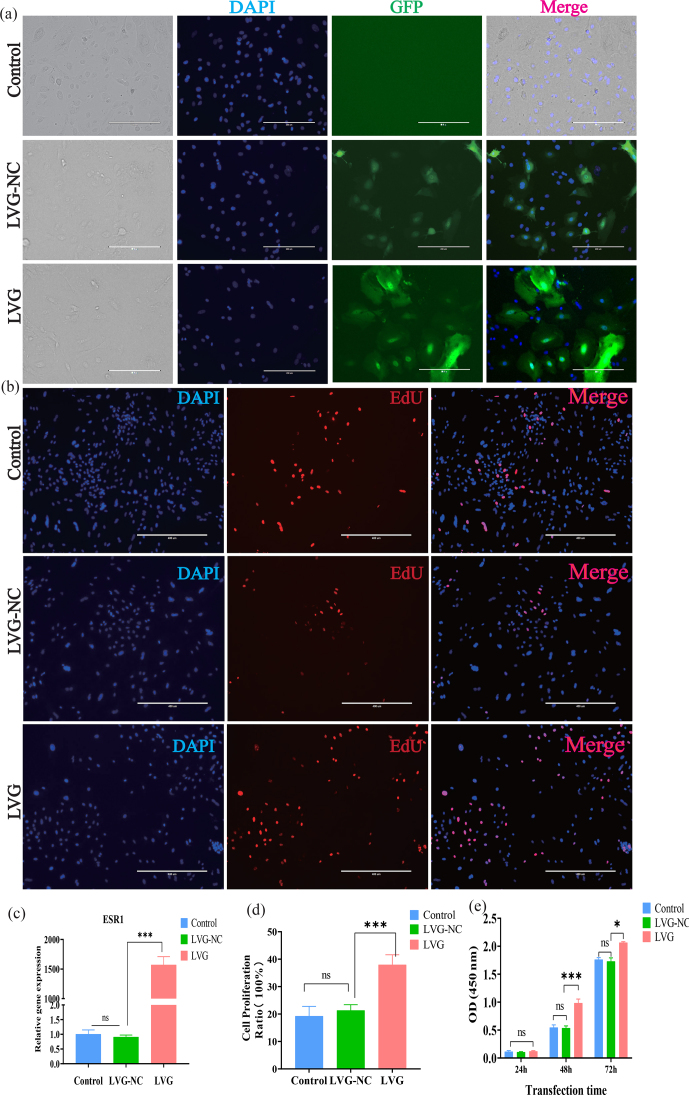
The effect of *ESR1* overexpression on the proliferation of buffalo mammary epithelial cells (BMECs). **(a)** Fluorescent detection of BMECs after lentivirus transfection; Scale=200µm . **(b)** EDU detection of BMECs after *ESR1* overexpression; Scale=400µm . **(c)** Detection of *ESR1* overexpression efficiency. **(d)** Statistical data of EdU detection. **(e)** CCK-8 detection of proliferation of BMECs after *ESR1* overexpression. Note: *, **, and *** represent p<0.05 , p<0.01 , and p<0.001 .

Green fluorescence was observed in BMECs 48 h after lentiviral infection, confirming successful vector delivery (Fig. 4a), indicating that *ESR1* was successfully transfected into the BMECs. qRT-PCR analysis showed that the expression level of *ESR1* in buffalo mammary epithelial cells was extremely significantly increased after transfection (Fig. 4c), suggesting that the *ESR1* overexpression vector functioned well. The EdU assay revealed that the viability of BMECs was significantly enhanced after *ESR1* overexpression (Fig. 4b and d). The CCK-8 assay showed that, compared with the control group, the OD values of buffalo mammary epithelial cells with *ESR1* overexpression were significantly increased at 48 and 72 h (Fig. 4e), indicating that *ESR1* overexpression promoted the proliferation of mammary epithelial cells.

### 3.5 Effect of *ESR1* overexpression on gene expression in BMECs

**Figure 5:**
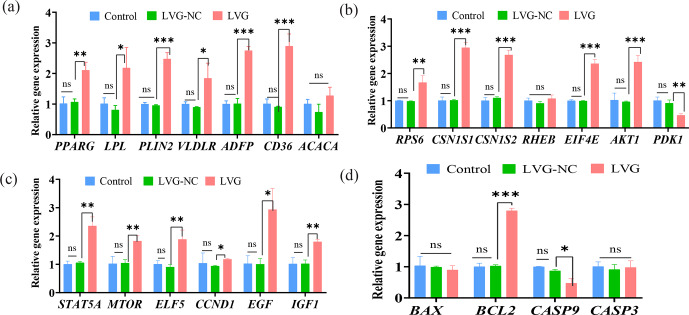
The effect of *ESR1* overexpression on gene expression in buffalo mammary epithelial cells (BMECs). **(a)** The effect of *ESR1* overexpression on the expression of genes related to milk fat synthesis. **(b)** The effect of *ESR1* overexpression on the expression of genes related to milk protein synthesis. **(c)** The effect of *ESR1* overexpression on the expression of genes related to cell proliferation. **(d)** The effect of *ESR1* overexpression on the expression of genes related to cell apoptosis. Note: *, **, and *** represent p<0.05 , p<0.01 , and p<0.001 .

qRT-PCR was further performed to investigate the effect of *ESR1* overexpression in BMECs on the transcription activities of milk synthesis and secretion traits. Results showed that overexpression of the *ESR1* gene significantly upregulated the expression of genes related to lipid transport and secretion, including *PPARG*, *LPL*, *PLIN2*, *VLDLR*, *ADFP*, and *CD36*, while the expression of *ACACA* did not change significantly (Fig. 5a). Further investigation into milk protein synthesis-related genes revealed that *ESR1* overexpression significantly increased the expression of *CSN1S1*, *CSN1S2*, *RPS6*, *RHEB*, *EIF4E*, and *AKT1* (Fig. 5b), indicating that *ESR1* overexpression can promote protein synthesis in BMECs. In addition, overexpression of *ESR1* significantly upregulated the expression of *STAT5A*, *MTOR*, *ELF5*, *CCND1*, *EGF*, and *IGF1* (Fig. 5c). Concurrently, the expression of *BAX* and *CASP9* was significantly decreased, while the expression of *BCL2* was significantly increased (Fig. 5d). These results suggested that *ESR1* overexpression can promote the proliferation of BMECs and inhibit apoptosis. The effects of *ESR1* overexpression on buffalo mammary epithelial cells were completely opposite to those of siRNA interference, further demonstrating the role of the *ESR1* gene in regulating substance synthesis and proliferation in buffalo mammary epithelial cells.

## 4 Discussion

Mammary gland development and lactation are tightly regulated by the endocrine system, with estrogen serving as a key regulator of postnatal mammary ductal branching (Biswas et al., 2022). Estradiol (E2) is primarily synthesized and secreted by the granulosa cells of developing ovarian follicles during the estrous cycle (Watson and Khaled, 2020). Estradiol (E2) signals primarily through estrogen receptors ER α (ESR1) and ER β . Accumulating evidence indicates that ESR1 is indispensable for normal mammary development and function, whereas ER β is largely dispensable (Rusidzé et al., 2021). Transcriptomic studies in dairy cows and mice further link dynamic *ESR1* expression to the maintenance of lobuloalveolar structure and milk component synthesis throughout lactation (Crisà et al., 2026). However, the specific regulatory mechanisms of *ESR1* in buffalo lactation have remained elusive.

In BMECs, the synthesis of substances such as milk fat and milk protein, along with cell proliferation and apoptosis, are key factors influencing lactation traits. The regulation of bovine milk component synthesis by *ESR1* involves both the direct impact on structural protein gene expression and the systemic modulation of metabolic pathways. Milk fat synthesis is an extremely complex and finely regulated physiological process, encompassing three tightly interconnected and indispensable parts: lipid uptake and transport, fatty acid synthesis, and lipid storage. During the lipid uptake and transport stage, mammary epithelial cells primarily rely on *LPL*-mediated hydrolysis of circulating lipoproteins, breaking down triglycerides in chylomicrons and very-low-density lipoproteins (VLDLs) into free fatty acids (FFAs) and glycerol for cellular uptake and utilization; *CD36* mediates the transmembrane transport of long-chain fatty acids (LCFAs), ensuring efficient acquisition of exogenous LCFAs, providing key raw materials for milk fat synthesis; *VLDLR* provides fatty acids and cholesterol to the cell via endocytosis of VLDLs. Studies have shown that *CD36* mRNA expression is significantly upregulated during the bovine lactation period (Bionaz and Loor, 2008), and *CD36* overexpression can increase cellular uptake of long-chain fatty acids (Ehehalt et al., 2008). These findings align with previous studies showing that E2-ESR1 signalling upregulates *CD36* and fatty acid uptake in muscle cells (Liu et al., 2019). In this study, after siRNA interference targeting the *ESR1* gene, *CD36*, *LPL*, and *VLDLR* expression significantly decreased, while overexpression showed the opposite trend, consistently with previous research findings, indicating that *ESR1* plays an important role in the lipid uptake process. Studies have shown that the *PPARG* gene plays a crucial role in regulating fatty acid synthesis. Specifically, knockdown of the *PPARG* gene significantly reduces the expression of genes related to milk fat synthesis (such as *CD36*) in buffalo mammary epithelial cells while simultaneously decreasing the triglyceride content. In contrast, overexpression of the *PPARG* gene remarkably increases the expression of these genes and the triglyceride content. These findings indicate that PPARG is a key gene regulating milk fat synthesis in buffalo (Zhou et al., 2021). Previous studies found that, in bovine mammary tissue, although *PPARG* mRNA abundance is low, its upregulation during lactation suggests a potential role in milk fat synthesis (Bionaz and Loor, 2008). As a key transcription factor in lipid metabolism, PPARG plays an indispensable role in milk fat synthesis (Wang et al., 2024). Previous studies have shown that *ESR1* can recruit the co-activator PPARGC1B to upregulate its expression. Because *PPARGC1B* acts as a crucial co-activator for *PPARG*, its enhanced expression significantly increases *PPARG* transcriptional activity (Li et al., 2011). Consistently with these findings, our study also demonstrated that *ESR1* significantly and positively regulates the transcription of *PPARG*. Furthermore, *PPARG* has been reported to upregulate the expression of *CD36* and *LPL* in mammary cells (Dao et al., 2025). In the present study, the expression trends of *CD36* and *LPL* were highly synchronized with those of *ESR1* and *PPARG*. Therefore, we hypothesize that *ESR1* may amplify the regulatory network of lipid metabolism pathways via *PPARG*. Lipid droplets, serving as the primary sites for triglyceride storage and secretion in mammary cells, rely on the regulation of the lipid-droplet-associated proteins *PLIN2* and *ADFP* for their stability (Yang et al., 2025). This study found that *ESR1* has a positive regulatory effect on both *PLIN2* and *ADFP*; their expression levels decreased after *ESR1* inhibition and increased at the mRNA level after overexpression. In adipocytes, researchers have found that the expression of perilipin proteins like *PLIN2* and *ADFP* is regulated by various nuclear receptors, including the estrogen-related receptor α (ERR α ). ERR α can directly bind to specific sequences (RE-3) in the *PLIN2* gene promoter region and regulate its transcription. This indicates that ERR α plays an important role in lipid droplet formation and maintenance (Akter et al., 2008). Therefore, we can speculate that *ESR1* may indirectly influence *PLIN2* expression via ERR α , thereby maintaining lipid droplet stability. This regulatory mechanism may also apply in mammary cells as stable lipid droplet structure is a prerequisite for milk fat synthesis and secretion.

Milk protein, primarily composed of casein and whey proteins, is a critical determinant of milk quality. Caseins can interact with each other and bind with calcium phosphate to form micelles, which play an essential role in preventing casein precipitation and stabilizing milk emulsion (Runthala et al., 2023). *CSN1S1*, *CSN1S2*, and *CSN2* belong to the casein gene family. As tissue-specific genes expressed in the mammary gland, they collectively participate in the synthesis and secretion of milk proteins, exert synergistic effects on the nutritional and physicochemical properties of milk, and are vital for the milk yield of water buffalo. However, a significant research gap remains regarding the specific mechanisms by which the *ESR1* gene regulates milk protein synthesis in BMECs. Using primary mammary epithelial cells from lactating dairy cows as a model, Yu et al. (2022) demonstrated that *ESR1* indirectly and positively regulates *CSN1S1* and *CSN1S2* and serves as an essential factor for daidzein-induced casein synthesis; specifically, daidzein dose-dependently upregulated CSN1S1 and CSN1S2 protein expression, an effect that was blocked upon *ESR1* knockdown by siRNA, thereby revealing that ESR1 indirectly upregulates *CSN1S1* and *CSN1S2* expression via the ER α –NF κ B1-mTOR cascade pathway. These findings are highly consistent with our results, which demonstrate that *ESR1* upregulates the expression of *CSN1S1* and *CSN1S2* (Yu et al., 2022). Furthermore, studies have established that the PI3K-AKT-mTOR pathway plays a pivotal regulatory role in the process of milk protein synthesis (Li et al., 2023). Our results revealed that *ESR1* significantly and positively regulates the expression of key genes within the mTOR signalling pathway, such as *RPS6*, *RHEB*, *EIF4E*, and *AKT1*; therefore, we hypothesize that *ESR1* influences milk protein synthesis by modulating the mTOR signalling cascade. Notably, the present study found that *ESR1* significantly and negatively regulates the expression of *PDK1*. Previous studies have identified *PDK1* as a key mitochondrial enzyme overexpressed in many cancer cells, which shifts glucose metabolism from oxidative phosphorylation toward aerobic glycolysis (Cenigaonandia-Campillo et al., 2021). Specifically, *PDK1* inhibits the activity of the pyruvate dehydrogenase complex (PDH) via phosphorylation, thereby diverting more pyruvate into the glycolytic pathway to generate lactate rather than entering the tricarboxylic acid (TCA) cycle. Therefore, it can be inferred that, in mammary epithelial cells, when high ESR1 expression induces a decrease in PDK1 levels, PDH activity is restored, allowing pyruvate to smoothly enter the TCA cycle. This shift in metabolic pattern effectively enhances cellular oxidative phosphorylation efficiency, thereby providing the ATP energy support and amino acid metabolic precursors required for the highly energy-consuming milk protein synthesis process driven by the mTOR signalling pathway, ultimately ensuring the efficient synthesis of milk proteins.

Furthermore, the viability and number of mammary epithelial cells are also crucial factors affecting milk yield. Dairy cows possess a larger alveolar area and higher cell numbers during peak lactation compared to other periods (Deacon et al., 2023). Increasing mammary cell numbers or reducing apoptosis can enhance lactation persistency. Therefore, an increase in the number of mammary secretory cells can, to some extent, improve the efficiency of milk synthesis. The *ESR1* gene, as a core component of the estrogen signalling pathway, plays a vital role in mammary development and lactation function. Researchers have pointed out that, in goat mammary epithelial cells, estrogen binding to *ESR1* promotes their proliferation. *ESR1* expression increases during pregnancy when mammary tissue begins to expand to facilitate mammary development (Ogorevc and Dovč, 2016). Moreover, mouse experiments have found that *Esr1* expression in mammary epithelial cells is essential for murine ductal and alveolar morphogenesis; loss of *Esr1* during the mouse pregnancy cycle leads to lobuloalveolar loss, impaired ductal side branching, and insufficient milk delivery (Feng et al., 2007). However, in buffalo, an important dairy species, there is currently a lack of systematic and in-depth exploration regarding the impact of the *ESR1* gene on mammary epithelial cell proliferation. In this study, siRNA interference targeting *ESR1* and lentiviral transfection for *ESR1* overexpression demonstrated that *ESR1* can promote the lactation process by regulating the expression levels of cell-proliferation-related genes. Furthermore, EdU and CCK-8 assays verified that *ESR1* has a significant effect on the proliferation of mammary epithelial cells. In our study, *ESR1* had a positive effect on BMEC proliferation and viability. After interfering with *ESR1*, the expression levels of the cell-proliferation-related genes *STAT5A*, *MTOR*, *ELF5*, *CCND1*, *EGF*, and *IGF1* were significantly downregulated, while the expression levels of the pro-apoptotic genes *BAX* and *CASP9* were significantly increased, and the expression level of the anti-apoptotic gene *BCL2* was significantly decreased. After overexpressing *ESR1*, the expression levels of *STAT5A*, *MTOR*, *ELF5*, *EGF*, and *IGF1* were significantly upregulated; the expression level of the anti-apoptotic gene *BCL2* was significantly increased; and the expression level of the pro-apoptotic gene *CASP9* was significantly decreased, while the expression levels of *CASP3* and *BAX* showed no significant change. *STAT5A*, as a key transcription activator, can regulate the expression of various cell-cycle-related genes, thereby driving cell proliferation. Studies have shown that, in mouse experiments, when mammary organoids were exposed to $1\times 10^{-9}\;M$ bisphenol A (BPA) and $1\times 10^{-12}\;M$ benzophenone-3 (BP3), *ESR1* mRNA expression increased, and *STAT5A* mRNA expression also increased (Altamirano et al., 2020), consistently with our findings. Furthermore, Edu and CCK-8 data indicated that, after interfering with *ESR1*, buffalo mammary epithelial cell proliferation slowed significantly, while proliferation accelerated significantly after overexpressing *ESR1*. This aligns with previous research results, further supporting the view that *ESR1* expression contributes to BMEC proliferation.

While the present findings underscore a positive regulatory role of *ESR1* in buffalo lactation, further investigations are still required to facilitate its practical application in molecular breeding. Profiling ESR1 expression patterns and identifying its functional genetic variants can provide valuable candidate molecular markers. Such markers can be integrated into marker-assisted selection (MAS) and genomic selection (GS) programmes, enabling the targeted screening and selective breeding of elite buffalo individuals with inherently high milk yield and improved milk composition traits. Nevertheless, the potential limitations of ESR1 modulation should not be overlooked. Artificial excessive activation of mammary epithelial cell proliferation and milk synthesis may induce metabolic stress and negative energy balance throughout lactation. In addition, given that ESR1 acts as a pleiotropic receptor with essential roles in systemic reproductive regulation, its aberrant overactivation could disturb endocrine homeostasis, thereby impairing reproductive performance and elevating the susceptibility to mammary gland disorders. Accordingly, further in vivo studies are warranted to define the optimal range of *ESR1* activity, which can maximize lactation performance while maintaining satisfactory physiological health and reproductive capacity in buffaloes.

## 5 Conclusions

The present study demonstrated that *ESR1* modulates lactation-related traits in buffalo mammary epithelial cells (BMECs) via three distinct regulatory mechanisms. First, *ESR1* upregulates lipid-transport-related genes including *PPARG* and *LPL*, thereby facilitating fatty acid uptake and transport, while exerting no obvious effect on de novo fatty acid synthesis. Second, it activates the MTOR signalling pathway by upregulating downstream genes such as *AKT1* and *RHEB*, which further enhances the expression of milk protein synthesis-associated genes. Third, *ESR1* elevates the expression of proliferation-related genes (e.g. *STAT5A*) and suppresses the levels of pro-apoptotic genes (*BAX* and *CASP9*), consequently promoting BMEC proliferation and inhibiting cellular apoptosis. Collectively, these findings provide an important theoretical basis for elucidating the molecular regulatory mechanism underlying buffalo lactation and offer valuable insights for future molecular breeding programmes.

## Supplement

10.5194/aab-69-347-2026-supplement The supplement related to this article is available online at https://doi.org/10.5194/aab-69-347-2026-supplement.

## Data Availability

All data generated and analysed during the present study are included in this published article. No additional unpublished data are available.
