# Supplementary material for: The role of ESR1 in regulating lactation traits in buffalo mammary epithelial cells
Source: Arch Anim Breed. 2026 Jun 25;69(2):347–61. doi: 10.5194/aab-69-347-2026 (PMC13379184; doi:10.5194/aab-69-347-2026)
Supplement: The supplement related to this article is available online at https://doi.org/10.5194/aab-69-347-2026-supplement. [file aab-69-347-2026-supplement.pdf]

Supplement of Arch. Anim. Breed., 69, 347–361, 2026
https://doi.org/10.5194/aab-69-347-2026-supplement
© Author(s) 2026. CC BY 4.0 License.

Open Access
Archives Animal Breeding

Supplement of

The role of *ESR1* in regulating lactation traits in buffalo mammary epithelial cells

Zhixiang Wang et al.

Correspondence to: Zhipeng Li (zp.li@gxu.edu.cn)

The copyright of individual parts of the supplement might differ from the article licence.

Supplementary file:

Figure S1: Exploration of optimal MOI conditions

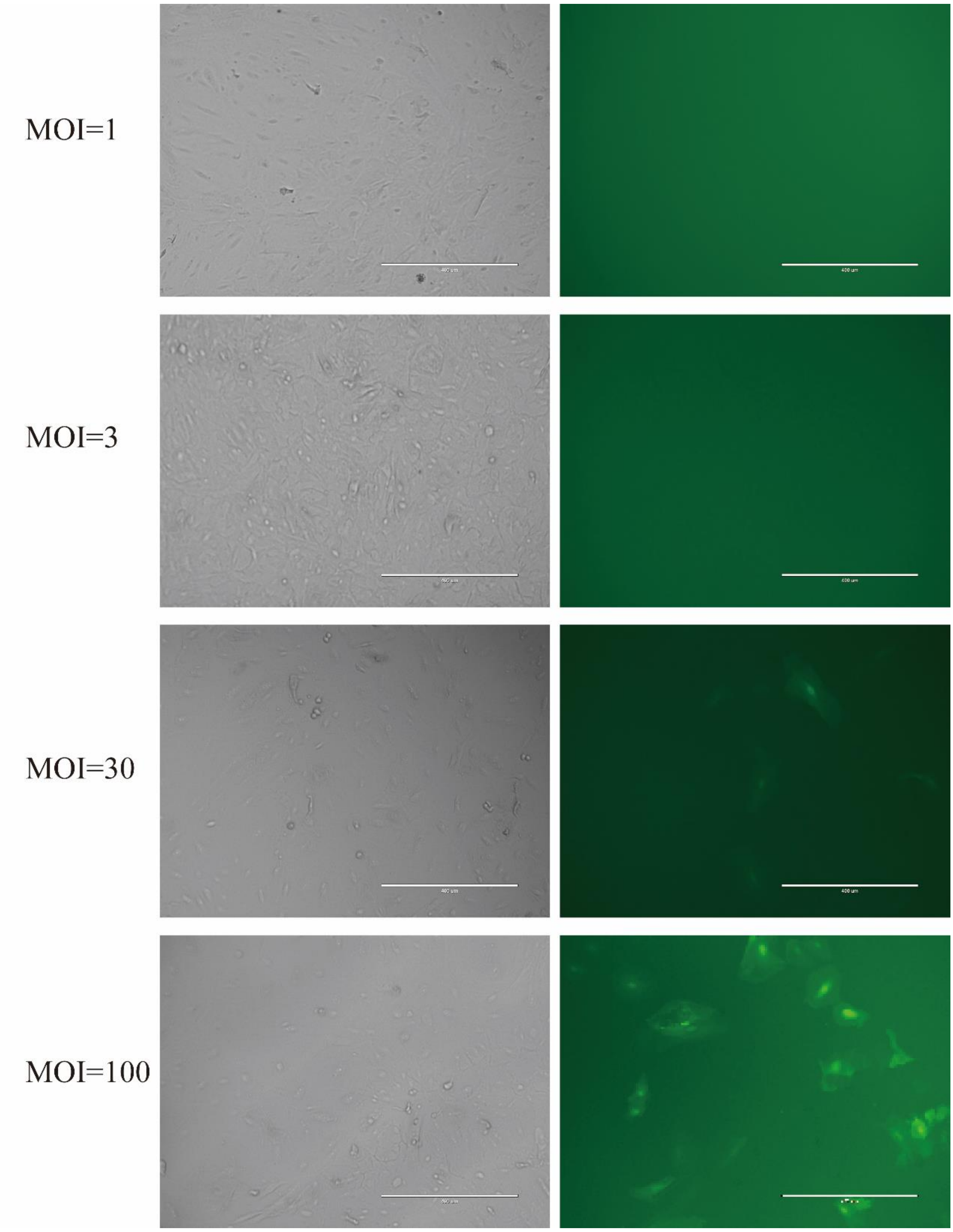

Fluorescent detection of buffalo mammary epithelial cells after lentivirus infection.
